# Calciphylaxis After Aortic Valve Replacement in a Patient with End-Stage Renal Disease

**DOI:** 10.3400/avd.cr.21-00040

**Published:** 2021-12-25

**Authors:** Kazunobu Hirooka, Keisuke Anju, Yoshihiro Moriyama, Yuichi Araki, Ekapot Bhunchet, Ryoji Kinoshita, Yohei Yamamoto, Hidetoshi Uchiyama, Masahiro Oonuki, Hiroyuki Tanaka

**Affiliations:** 1 Department of Cardiac and Vascular Surgery, Tsuchiura Kyodo General Hospital, Tsuchiura, Ibaraki, Japan; 2 Department of Dermatology, Tsuchiura Kyodo General Hospital, Tsuchiura, Ibaraki, Japan; 3 Department of Emergency, Tsuchiura Kyodo General Hospital, Tsuchiura, Ibaraki, Japan; 4 Department of Pathology, Tsuchiura Kyodo General Hospital, Tsuchiura, Ibaraki, Japan

**Keywords:** calciphylaxis, end-stage renal disease, aortic valve replacement

## Abstract

In this study, we report a case of a patient on dialysis who presented necrotic lesions on the legs and penile ulceration 7 years after a mechanical aortic valve replacement. The diagnosis of calciphylaxis was not confirmed even after skin biopsy, and multidisciplinary management was not initiated until the patient was admitted with septic shock. Cardiovascular surgeons should be aware of warfarin-induced calciphylaxis, whose pathophysiology differs from that of atherosclerosis. Considering poor long-term survival of dialysis patients, mechanical valves should be reserved only for those patients whose estimated survival is longer than the time taken for a biological valve to deteriorate.

## Introduction

Calciphylaxis has been characterized by progressive skin necrosis associated with severe painful skin lesions. Necrotic skin lesions demonstrate poor healing and are frequently complicated by blistering and ulceration with superimposed infections. This disease has been recognized as a disorder of microvascular calcification and thrombosis; it predominantly affects patients with end-stage renal disease (ESRD) who undergo hemodialysis.^1)^ The risk factors that predispose patients to calciphylaxis are ESRD, more than 50 years of age, hypoalbuminemia, hypercalcemia, diabetes, connective tissue disease, hypercoagulable disorders, medications including calcium-based phosphate binders, vitamin K antagonists (warfarin), corticosteroids, and subcutaneous injections of insulin or heparin.^1–3)^ Warfarin administration is considered to be an important risk factor for the onset of this disease. Although the pathogenesis of warfarin-associated calciphylaxis is yet to be completely understood, evidence suggests that warfarin promotes vascular calcification by inhibiting the vitamin K-dependent matrix Gla protein that prevents calcium deposition in the arteries.^4)^

There is still some controversy among cardiac surgeons as to the optimal type of prosthetic valve for patients aged 50–65 years with ESRD who are undergoing hemodialysis. Most surgeons expect its long-term durability from mechanical valves, despite the potential risk of bleeding due to warfarin therapy. In contrast, biological valves have been presumed to undergo accelerated degeneration that causes the structural valve failure. In this study, we describe a patient with calciphylaxis who underwent hemodialysis with warfarin anticoagulation therapy for 7 years after valve replacement surgery.

## Case Report

A 64-year-old man with ESRD undergoing peritoneal dialysis was admitted to our center in June 2012 for severe aortic stenosis due to a bicuspid aortic valve. Valve replacement surgery was then performed with a mechanical valve (St. Jude Medical Regent 19-mm valve; St. Jude Medical, Minneapolis, MN, USA) due to the patient’s preference for its durability. The postoperative course was uneventful, and the oral anticoagulation therapy with warfarin was maintained at the outpatient clinic.

In March 2015, the peritoneal dialysis was changed to hemodialysis due to a marked increase in right pleural effusion with persistent hypoalbuminemia. In December 2018, the patient complained of bilateral calf pain after 50-m ambulation. The arterial blood pressure of both legs was almost normal at this time.

In June 2019, the patient was unable to walk due to severe calf pain. A pulsatile blood flow was clearly detected using Doppler ultrasound on both dorsalis pedis and posterior tibial arteries. Computed tomography-angiography showed multiple calcifications from the abdominal aorta to the distal arteries including small vessels on the body surface such as the penile artery. Blood flow below the knees was difficult to evaluate. During the physical examination, the patient was noted to have extensive gangrenous necrotic lesions on both calves and ulceration on the glans of the penis. Considering that the percutaneous oxygen pressure of the toes was maintained in both feet, calciphylaxis was suspected rather than arteriosclerosis obliterans. The dermatology department then performed a skin biopsy wherein non-specific histological changes compatible with skin necrosis were found.

**Figure 1:**
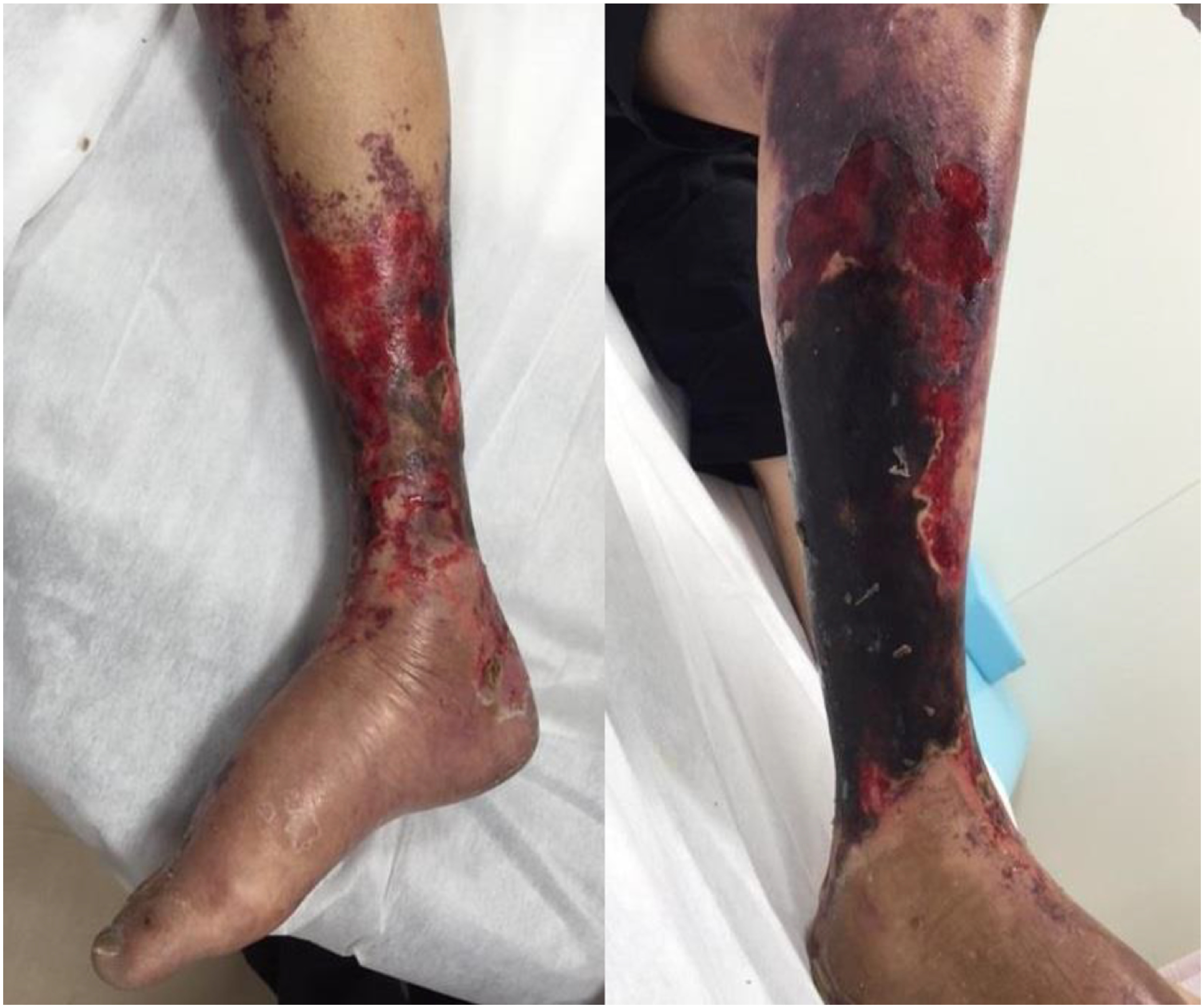
Fig. 1 Necrotic skin lesions demonstrating black eschar on the right lower leg.

In August 2019, the patient was admitted with tachypnea and altered mental status. The physical examination revealed an arterial blood pressure of 91/40 mmHg, pulse rate of 81 bpm, respiratory rate of 34 bpm, and oxygen saturation of approximately 90%. The necrotic leg lesions extended and penetrated into the muscular fascia of both calves (**Fig. 1**).

The laboratory results were as follows: C-reactive protein, 34 mg/dL; creatinine, 5.71 mg/dL; blood urea nitrogen, 50 mg/dL; calcium, 9.4 mg/dL; hemoglobin, 10.3 g/dL; white blood cells, 19000/µL; platelets, 1.2×10^4^/µL; and albumin, 2.2 g/dL. Warfarin was then discontinued on presentation because of the excessive anticoagulant state (prothrombin time-international normalized ratio >9.9).

**Figure 2:**
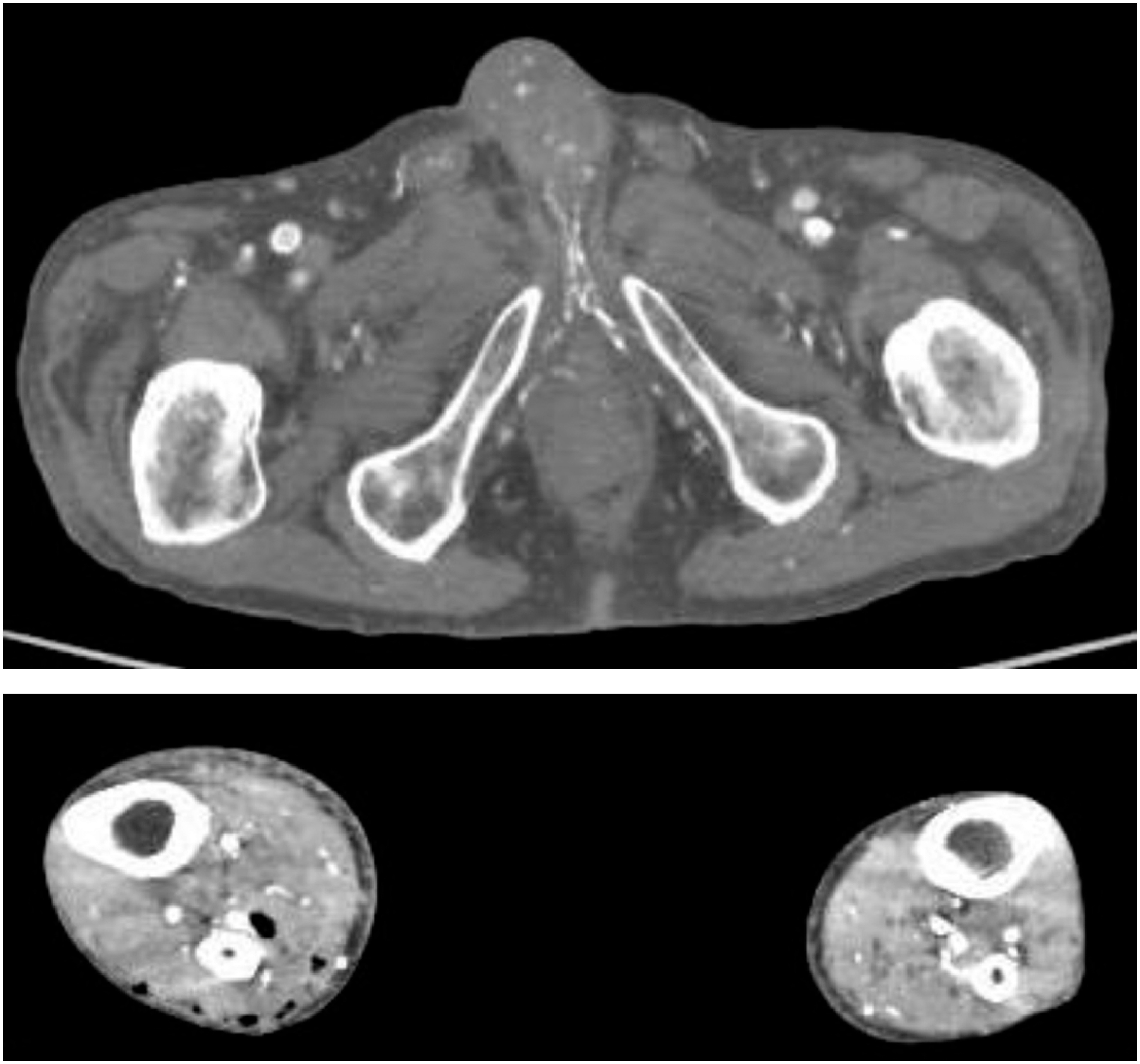
Fig. 2 Computed tomography showing penile and femoral artery calcifications. Gaseous cellulitis is noted in the right lower leg.

Computed tomography revealed massive bilateral pleural effusion and multiple air bubbles in the subcutaneous tissue and muscles of the right lower leg (**Fig. 2**).

**Figure 3:**
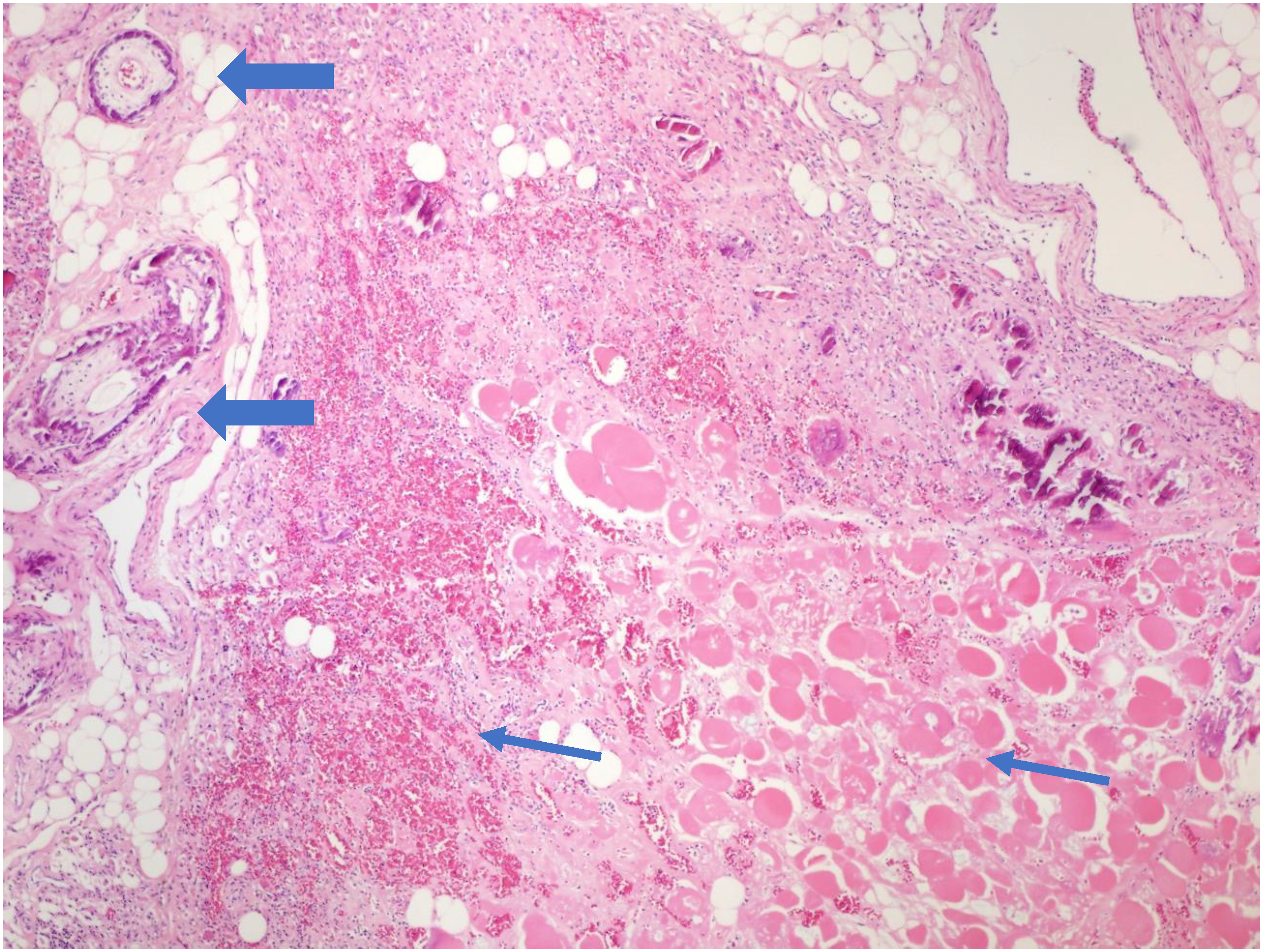
Fig. 3 Histological examination revealed severe stenosis with medial calcification and intimal proliferation in small arteries (thick arrows). Necrotic muscles were observed with congestion and bleeding (thin arrows).

Given the patient’s diagnosis of septic shock due to gas gangrene in the right leg, we performed an emergent amputation above the knee and debridement of the necrotic lesions of the left calf. His postoperative hemodynamic status remained unstable and required vasopressors. Although the antibiotic administration of vancomycin and tazobactam was started, the wound infection in the stump has rapidly exacerbated, and the patient expired after 7 days of intensive care. The diagnosis of calciphylaxis was confirmed via histological findings, showing the medial calcification and the intimal proliferation, with microthrombi in the small vessels, which were obtained from the specimen of the amputated leg (**Fig. 3**).

## Discussion

The true incidence of calciphylaxis remains unclear and has been difficult to ascertain using administrative data due to lacking unique International Classification of Diseases (ICD) 10th Revision code. Data from a nationwide registry in Germany in 2017 estimated an annual incidence rate of 0.04% in German dialysis patients.^5)^ Among them, the 1-year mortality for patients with calciphylaxis is reported at 45%–80%, with ulcerated lesions associated with higher mortality compared to non-ulcerated lesions and sepsis being the leading cause of death.^1)^

Calciphylaxis is primarily a clinical diagnosis, obtained by the presence of livedoid skin changes or ulcers, often with a thick eschar overlying painful indurated subcutaneous plaques located predominantly in adipose-rich areas.

A skin biopsy is necessary to confirm the diagnosis. Typical histologic features in calciphylaxis include dermal and extravascular or pannicular small-vessel calcification and thrombosis, along with intimal fibrosis. However, such findings had low sensitivity and low specificity, and the utility of a biopsy was questionable.^2)^ Moreover, skin biopsy might result in the exacerbation of the ulcer, infection, and even propagation of new lesions. Thus, there is a need for further research in this area and for a proper biopsy technique.^2,6)^

This present case could have been diagnosed earlier as the patient had been exhibiting two risk factors, that is, ESRD on hemodialysis and warfarin therapy for the mechanical aortic valve.

In addition, we noted upon a review of the medical history that the patient had experienced a penile ulcer 6 months prior to admission. Authors have reported that ischemic necrosis or ulceration of the penis has been recognized as proximal calciphylaxis, caused by calcified changes in small arteries originating from the internal pudendal artery, a branch of the internal iliac artery. Most patients with proximal calciphylaxis have dismal prognoses.^4)^

There are no consensus guidelines on the management of calciphylaxis. The reported treatment generally involves a multi-interventional and multidisciplinary approach, including wound care, pain management, administration of sodium thiosulfate in dialysis solutions, cinacalcet for bone mineral disease, hyperbaric oxygen therapy, and discontinuation of offending medication, particularly warfarin.^1)^ In this present case, alternate anticoagulation options were limited because the mechanical aortic valve was implanted. Switching the drug to enoxaparin after bridging to low-molecular-weight heparin is deemed a controversial decision.^1,6)^

Mortality rates among dialysis patients remain to be high. According to the data extracted from the US Renal Data System in 2017, the 5-year survival rate for patients with ESRD on dialysis was approximately 40%. Cardiovascular diseases have been identified as the leading cause of death in this patient population.

It is believed that biological valves rapidly calcify in patients undergoing dialysis. Kaplon et al.^7)^ analyzed 42 patients undergoing dialysis, of which 25 received biological valve replacement and 17 received mechanical valve replacement surgery. The estimated 5-year survival rate was 27% for patients with biological valves, whereas it was 33% for those with mechanical valves, and there was no significant difference in terms of survival between them. In their series, reoperation was required for one patient (5%) due to calcific degeneration of the biological valve. Brinkman et al.^8)^ reported structural dysfunction of a biological valve in 2 (7%) of the 30 patients who were undergoing chronic renal dialysis within 15 months and 54 months of implantation, respectively. Despite anecdotal reports, accelerated calcification of biological valves in patients on dialysis remains uncommon. Manghelli et al.^9)^ reviewed 423 dialysis-dependent patients, of which 341 had biological and 82 had mechanical valves. The 5-year survival rates were 23% and 33% for the biological and mechanical groups, respectively. After adjusting for age, NYHA (New York Heart Association) class, and diabetes, survival was similar between the groups. Cox regression analysis was also performed to estimate survival based on five different ages (30, 40, 50, 60, or 70 years old) and the presence of diabetes and/or heart failure. They showed that only patients aged 30 or 40 years with NYHA class I–II failure without diabetes had a >50% estimated 5-year survival rate. Because anticoagulation carries an increased risk of morbidity, they suggested that mechanical valves should be reserved only for those patients with an estimated long-time survival, i.e., longer than the time a biological valve may deteriorate; this includes very young people (e.g., 30 or 40 years old) without diabetes or NYHA class III or IV symptoms.

In summary, further research is needed to understand the pathogenesis and identify the risk factors for calciphylaxis. Clinicians, including cardiovascular surgeons, should be aware of the clinical characteristics of calciphylaxis and the therapeutic dilemma of using warfarin and maintain a high index of clinical suspicion for an early and accurate diagnosis.

## Conclusion

Warfarin use is relatively safe in the treatment for various diseases. However, considering its rare but serious complication of calciphylaxis, more caution should be observed when using warfarin. Also, greater sensitivity in identifying ischemic skin lesions, particularly in patients with ESRD on hemodialysis, is required. An early diagnosis of calciphylaxis is crucial for starting accurate multidisciplinary treatment, including the discontinuation of warfarin. Since hemodialysis-dependent patients who undergo valve replacement surgery have poor long-term survival, biological valves should be strongly considered to avoid further morbidities due to mechanical valves.
